# Species annotation using a k-mer based KNN model

**DOI:** 10.6026/973206300200986

**Published:** 2024-09-30

**Authors:** Srushti Sangar, Prathamesh Kolage, Pritee Chunarkar-Patil

**Affiliations:** 1 Department of Bioinformatics, Rajiv Gandhi Institute of IT and Biotechnology, Bharati Vidyapeeth (Deemed to be University), Pune, Maharashtra, India

**Keywords:** k-mer, bacterial identification, sequence comparison, KNN classification, bio pytho

## Abstract

Bacterial identification is a critical process in microbiology, clinical diagnostics, environmental monitoring, and food safety. Machine learning holds great promise for improving bacterial identification by increasing accuracy, speed, and scalability. However, challenges such as data dependency, model interpretability, and computational demands must be addressed to fully realize it's potential. k-mer based bacterial identification algorithm is an attempt to address these issues. Sequence matching is completed using the KNN technique. This included feature extraction, dataset preparation, classifier training, and label prediction based on k-mer frequency distribution similarity. The algorithm's performance has been cross-checked through accuracy assessment metrics such as F1 score and precision with an impressive 93% accuracy rate.

## Background:

The annotation and identification of genomic regulatory elements, such as enhancers, splice sites, transcription start sites, and promoters, as well as the classification of various phenotypes, are just a few of the genomics and bioinformatics issues that have been extensively addressed by machine learning algorithms [1]. Utilizing the 16S rRNA gene as a pattern-based computational tool for taxonomy classification from the phylum Firmicutes down to the genus Bacillus, DNA Barcode Identification (DNA Bar ID) has recently generated a lot of interest in the use of k-mer-based methods to predict the phenotypic features of bacteria. The researchers mapped the patterns into several hyperactive variable areas of the 16S rRNA gene, with V3-V4 being one of the most highly variable regions. The produced signatures displayed good sensitivity and specificity when compared [2]. K-mer-based models may be easier to interpret if techniques for decreasing redundancy and collecting genomic context are employed. Evaluation of prediction models constructed with sparse machine learning techniques, such as decision trees or lasso-regression, can be more difficult when bacterial genomes are represented using k-mers. This is because, although there might be more linked features that are equally predictive, these algorithms usually select a random subset of the connected features [3]. Bio Seq-Analysis is a robust platform for biological sequence analysis that leverages machine-learning techniques [4]. It streamlines feature extraction, predictor development, and performance evaluation, automates prediction creation while allowing users to contribute benchmark datasets, and outperforms some state-of-the-art methods in sequence analysis tasks. Virus sequences may now be identified from prokaryotic metagenomics data thanks to tools such as VirFinder, which improve and supplement gene-based methods for viral sequence categorization. These methods provide an effective way to find new viruses that might not share gene sequences by utilizing virus-specific k-mer patterns. The efficient and dependable classification of viral sequences over a broad variety of host domains and phyla is made possible by the use of k-mer patterns in place of gene-centric approaches for viral sequence identification [5]. K-mers are brief segments of a predetermined length (k) that are taken out of DNA sequences [6]. Many methods of sequence classification have been proposed, with the goal of improving BLAST accuracy with machine learning and sequence matching algorithms. The MEGAN program searches a sequence (using BLAST) against many databases [7]. The lowest common ancestor (LCA) of the best matches discovered in each database is assigned to the sequence. To achieve higher accuracy than BLAST on its own, PhymmBL [8, 9] combines BLAST results with scores derived from interpolated Markov models. The Naïve Bayes Classifier (NBC) applies a Bayesian rule to the distributions of k-mers inside a genome [10]. Therefore, it is of interest to describe a k-mer based model using KNN.

## Materials and Methodology:

Through the integration of KNN algorithm and k-mer analysis, the tool facilitates fast and precise DNA sequence comparison for the identification of bacteria. This method increases the efficacy and efficiency of sequence matching in a variety of applications by utilizing both the natural properties of DNA sequences and the capabilities of machine learning. This program created to using the scikit-learn Tkinter, and bio-python libraries. The modular design allows for easy maintenance, scalability, and future enhancements.

## Data gathering and pre-processing:

DNA sequences were collected from various sources in order to build our reference database. These sequences span many organisms and genetic regions to ensure robustness in sequence comparison. We used the Biopython package to process FASTA files in an effective manner.

## Sequence comparison algorithm:

Our methodology is based on k-mer matching, a popular bioinformatics technique for sequence analysis. K-mers are short sub sequences of length 'k' that are extracted from DNA sequences. For each query sequence, we calculate the number of shared k-mers between the query and reference sequences.

## K-mer calculation:

A technique has been developed to extract k-mers from DNA sequences with efficiency. The function returns every possible k-mer of length 'k' given a sequence. These k-mers serve as the basis for the comparison between the reference and query sequences.

## Similarity calculation:

The degree of similarity between each reference sequence and the query sequence was measured by the fraction of shared k-mers. This percentage indicates how similar the sequence is to one another. Greater percentages imply greater similarity.

## User interface design:

**Figure 1:**
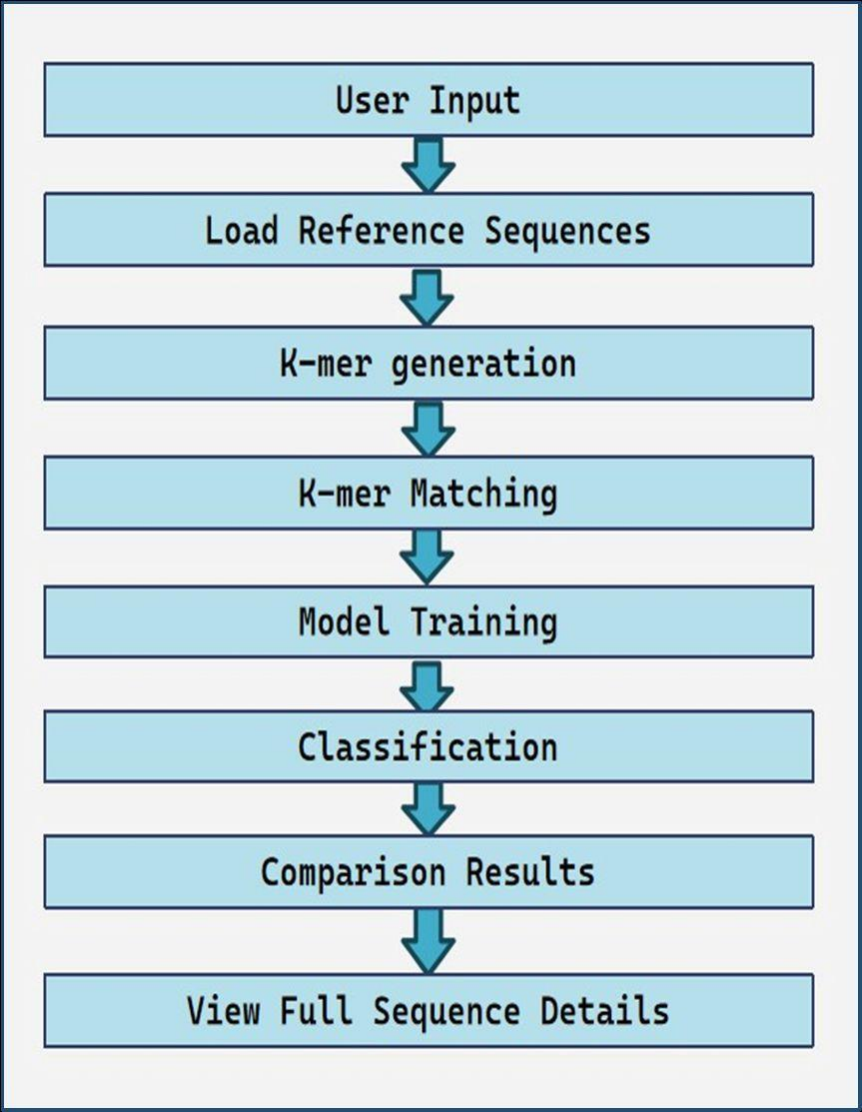
Flowchart of workflow of the tool

An easy-to-use graphical user interface created with the Python Tkinter module. Users can input their query sequences, start the comparison process, and adjust settings like the length of the k-mer and the number of k-mers to display with only one click. The complete workflow of the tool is shown in Figure 1.

## Results:

**Figure 2:**
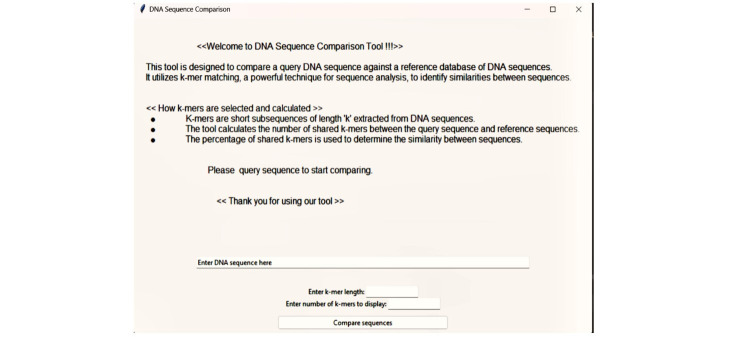
GUI of Tool

**Figure 3:**
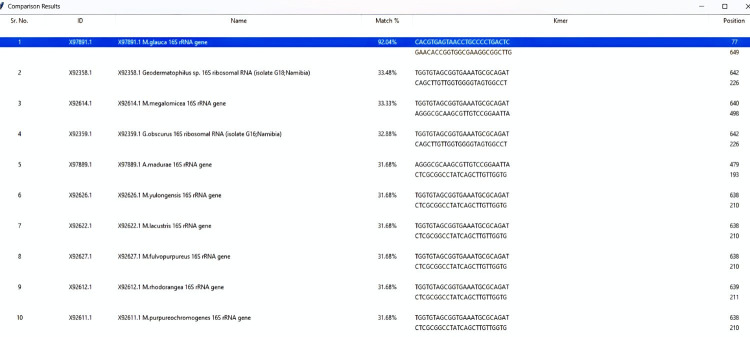
Results showing top 10 hits

**Figure 4:**
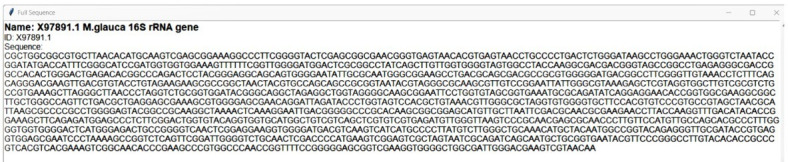
Sequence for one of the hits is displayed

The KNN method was selected for sequence matching in our model due to its resilience, simplicity, and efficiency in classification tasks. KNN is non-parametric and depends only on feature vector similarity, in contrast to parametric approaches that assume certain aspects of data distributions. This makes it especially appropriate for this study objective, which is to categorize query sequences according to how similar they are to reference sequences. k-mer-based prediction model provides a strong and effective method for identifying bacteria through the integration of KNN algorithm and k-mer analysis. This tool offers a flexible platform for quick and precise DNA sequence comparison. Users are able to change the number of matching k-mers that will be shown in the results as well as the k-mer length (k). By customizing the analysis parameters to the unique properties of their data and research goals, researchers can improve the relevance and usefulness of their findings. The homepage of the tools is shown (Figure 2). The tool will take sequence from the user and show the best 10 hits from the database (Figure 3). User can access the sequence through cross-link of accession number (Figure 4). The tool shows the percent match and position of the matched k-mer. The astounding 93% accuracy rate of our system was validated by metrics like precision and F1 score. Especially, considering the increasing application of machine learning in genomics and bioinformatics, our k-mer based prediction model stands out for its computational efficiency compared to traditional alignment approaches. Our model swiftly and precisely detects bacterial species using k-mer matching from DNA sequences. Python modules like Biopython and collections power it.

## Discussion:

Current study significantly enhances our understanding of k-mer-based prediction models in bioinformatics and genomics. It demonstrates that k-mer matching is an effective method for classifying DNA sequences, achieving a 93% accuracy rate in this prediction model. Unlike popular programs like Kraken and Mash, which focus on taxonomic classification or k-mer counting, current study approach prioritizes direct sequence comparison, offering researchers a novel paradigm [3, 11]. The model's flexibility allows users to create custom reference databases, making the analysis more relevant to specific research questions. This adaptability sets this method apart from technologies that rely on large, pre-built databases, enabling its application to a broader range of datasets. Our program also employs exact k-mer matching, which is crucial for accurate genomic research, providing detailed assessments of sequence similarities, including specific matching k-mers and their positions [12, 13]. Additionally, the user-friendly interface, developed with Tkinter, enhances accessibility and allows for interactive visualization of results, promoting the wider adoption of advanced genetic analysis techniques and democratizing the use of bioinformatics tools.

## Conclusion:

The k-mer-based prediction model represents a significant advancement in sequence comparison techniques, opening new possibilities for its application in various biological contexts [14]. The tool is having 93% accuracy, 96% positive predictive value, 91% sensitivity, 96% specificity and 90% negative predictive value (NPV). This study addresses current challenges and provides practical solutions for researchers, contributing valuable insights to genomics.
